# Bacterial Contamination of Medical Doctors and Students White Coats at Kilimanjaro Christian Medical Centre, Moshi, Tanzania

**DOI:** 10.1155/2015/507890

**Published:** 2015-11-04

**Authors:** Josephat Qaday, Margaretha Sariko, Adam Mwakyoma, Emmanuel Kifaro, Dominick Mosha, Richard Tarimo, Balthazar Nyombi, Elichilia Shao

**Affiliations:** ^1^Kilimanjaro Christian Medical University College, P.O. Box 2240, Moshi, Tanzania; ^2^Kilimanjaro Christian Medical Centre, Clinical Laboratory, P.O. Box 3010, Moshi, Tanzania; ^3^Kilimanjaro Christian Medical Centre Molecular Diagnostic Unit, P.O. Box 3010, Moshi, Tanzania; ^4^Ifakara Health Institute, P.O. Box 78373, Mikocheni, Dar es Salaam, Tanzania; ^5^Better Human Health Foundation, P.O. Box 1348, Moshi, Tanzania; ^6^Imagedoctors International, P.O. Box 16341, Arusha, Tanzania

## Abstract

*Background*. Microbial transmission from patient to patient has been linked to transient colonization of health care workers attires. Contamination of health care workers' clothing including white coats may play a big role in transmission of microbes. *Study Objective*. This study was conducted to determine the type of bacterial contamination on the white coats of medical doctors and students and associated factors. *Methods*. A cross-sectional study with purposive sampling of the bacterial contamination of white coats was undertaken. Demographic variables and white coats usage details were captured: when the coat was last washed, frequency of washing, washing agents used, and storage of the white coats. Swabs were collected from the mouth of left and right lower pockets, sleeves, and lapels of white coat in sterile techniques. *Results*. Out of 180 participants involved in the current study, 65.6% were males. Most of the coats were contaminated by staphylococci species and other bacteria such as Gram negative rods. *Conclusion and Recommendations*. White coats are potential source of cross infection which harbour bacterial agents and may play a big role in the transmission of nosocomial infection in health care settings. Effort should be made to discourage usage of white coats outside clinical areas.

## 1. Introduction

Clinical white coats have very long history of being a symbol of hope and healing for medical professionals; however there has been a concern that white coats may play a big role in transmitting infections within and outside hospital settings [1–4]. Wearing white coats by medical professional is accepted practice, but when, where, and how we wear and wash them vary among individuals and even between different institutions [1]. Patients-to-patients transmission of infections within health care facilities has been associated with transient harbouring of pathogens in health care workers and students clothing including white coats [5]. It is very common to see health care workers and students wearing white coats outside clinical areas such as canteen, supermarkets, library, and even the chapels [6]. It is also very common to see people hanging their white coats in their cars and offices or carrying them around outside hospital areas which increases chances for trafficking both pathogenic and nonpathogenic bacteria. Some of those bacterial strains might be resistant strains such as Methicillin Resistance* Staphylococcus aureus* (MRSA) which might be spread from hospital to the community and vice versa [7]. There are conflicting results about bacterial contamination of clinical white coat from USA (which concluded that they might be contaminated with pathogenic and resistant bacteria) and recent UK studies (which concluded that they may not be major culprit in the spread of nosocomial infection) [5–8]. Very little from African countries has been published on the issue of health care provider clothing and potential for contamination [9, 10]. In Tanzania, there is no documented report about the risks of white clinical coats towards spreading nosocomial infection. Therefore this study was conducted to determine bacterial contaminants present on clinical white coats of both medical doctors and students and factors associated with its contamination at KCMC referral and teaching hospital in Moshi, Tanzania. It was also aimed at ironing out conflicting findings between USA and UK studies on the risks of white coats contamination in health care settings. Furthermore we wanted to assess the hygienic use of these white coats such as cleaning, storage, time of wearing, and carriage as well as information on wearing them outside clinical areas.

## 2. Methods

Specimens were collected from left and right lower mouth pockets. Swabs were collected from the mouth of left and right lower pockets, sleeves. and lapels of white coats. The participants were instructed on how to take part during sample collection, and when he/she accepts to be included in the study, she/he signed the consent form and then the sample was taken. Sample collection was done using sterile swabs soaked with sterile normal saline, which was rubbed up and down or transverse at the left and right mouth pockets, sleeves, and lapels of the white coat (Figure 2). The collected sample was placed in a transport medium and transported to the laboratory for inoculation into Blood agar and MacConkey agar which then was incubated at 37°C overnight; the isolated microorganisms underwent different biochemical test to isolate type of microorganism such as catalase, coagulase, indole, oxidase, urea, and Kligler Iron Agar (KIA). Gram stain was also performed to confirm the bacterial characteristics, that is, Gram positive or Gram negative bacteria. All these procedures were performed following KCMC Clinical Laboratory standard operating procedures (SOPs).

## 3. Results

### 3.1. Social Demographic Characteristics of Participants

A total of 180 participants were enrolled in this study. Out of one hundred and eighty participants involved in the study 118 (65.6%) were males. About 60 (33.33%) were medical doctors and 120 (66.67%) were medical students. More participants were from nonsurgical departments, 100/180 (55.56%), while the rest were from surgical department. One hundred and fifty of the study participants (83.83%) were stationed at inpatients departments compared to the rest who were located at the outpatient department (Table 1).

### 3.2. Prevalence of Contamination

One hundred and thirty-two (73.33%) out of 180 whites coats were contaminated with different pathogens. The most dominant ones were* S. aureus*, 120 (91.67%),* Pseudomonas aeruginosa*, 9 (6.82%), and* E. coli*, 3 (2.27%) (Table 3).

## 4. Discussion

**Figure 1:**
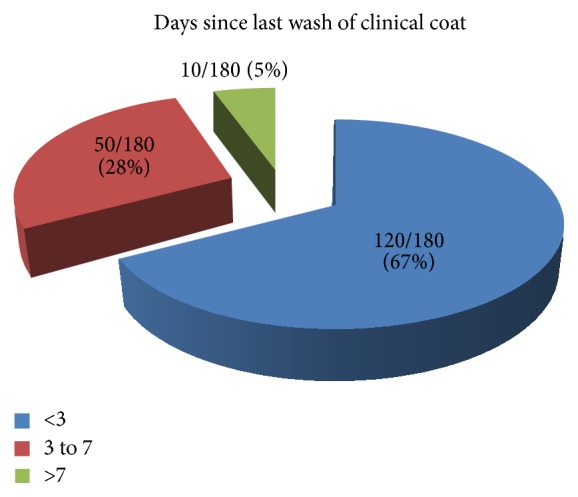
Days since last wash of clinical white coat (*N* = 180).

White coats traditionally represent dignity to medical professionals as well as hope and healing to patients [8]. However, these attires might carry serious pathogens which might lead to morbidity and mortality for both medical professionals and patients [11]. This may be partially explained by patient's continuous shading of microbes in hospital environment including health care workers who are constantly in contact with patients. In this study we had high prevalence of bacterial contaminants of 73.33% present in clinical white coats of medical doctors and students. We had more students with contaminated coats than doctors which might be due to students inexperience compared with qualified medical doctors; this may also be explained by methodology used which was convenient. As there is no special training for medical students on prevention of nosocomial infections, presence of students might increase risks for nosocomial infection in teaching hospitals. These findings were almost similar to another study in Columbia of 75% bacterial contamination and different from another study in Nigeria where contamination was 91.30% [9, 10, 12]. The higher prevalence in Nigeria compared to our study might partially be explained by the fact that our study was conducted at tertiary and referral hospital compared to Nigeria study which was conducted in the regional hospital.* S. aureus* was the major pathogen isolated (46.20%) which is also similar to other studies [1, 6, 12]. The high rate of contamination may include MRSA which are difficult to treat and hence increase costs of hospital stay, morbidity, and mortality unnecessarily. Our results were differently found by Uneke and Ijeoma where the most predominant isolated organisms were diphtheroids. This difference might be explained by different study population as well as geographical difference. Most of the white coats in this study were used less than 3 days before another round of washing (132/180) (73.33%) (Figure 1). Of all participants, white coats harboured bacteria from the mouth of left and right lower pockets, sleeves, and lapels of white coat. There was an association between duration of wearing white coats and severity of contamination but it was not significant (Table 2). The level of bacterial contamination in the current study was similar to a study in Nigeria by Uneke and Ijeoma which showed that about 91.3% of white coats had bacterial contamination mostly by diphtheroids followed by* Staphylococcus aureus*. This percentage is bigger compared with our study, but the difference might partially be explained by different geographical location as well as different level of clinical facilities in which the study was conducted. Our study was at the tertiary level where control of infection might be better than regional level which was the case in Nigeria's study [9].

Most of the coats were contaminated by staphylococci species and other bacteria such as Gram negative rods (Bacilli).* Staphylococcus aureus* (121/132) (91.67%) was the most common isolates, followed by* Pseudomonas aeruginosa* (9/132) (6.82%) and* E. coli* (3/132) (2.27%). Our study was in line with study conducted in Nigeria by Banu as well as Muhadi et al. in Malaysia which showed that* Staphylococcus aureus* was the most common bacterial contamination of the white coats of health care workers followed by coagulase negative staphylococci [6, 13]. Study conducted by Treakle et al. at University of Maryland School of Medicine showed that* Staphylococcus aureus* was dominated mostly in residents working in inpatients settings [5]. The staphylococci species are the most organisms isolated by every researcher especially* Staphylococcus aureus* [1]. It seems that* Staphylococcus aureus* was the common organism found in each study even in KCMC Hospital, Moshi, Tanzania, whereby the current study supported the previous findings.

**Figure 2:**
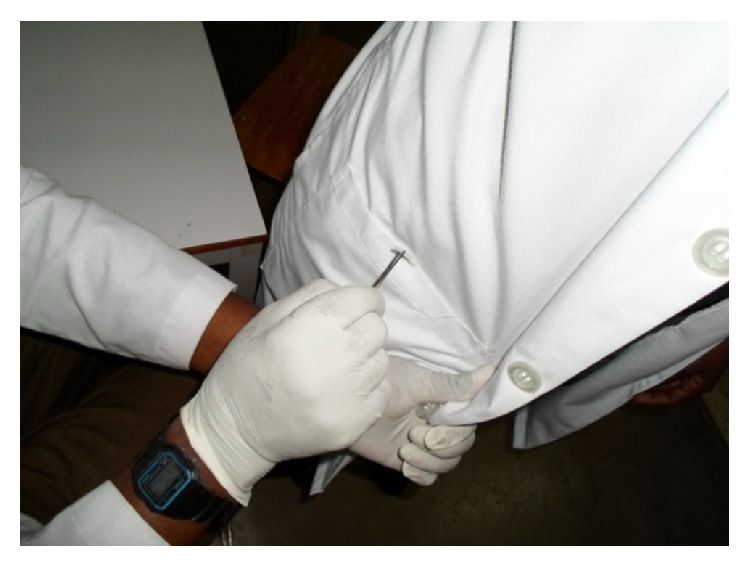
Techniques for swabbing white coats.

Most of our study participants were males (65.6%) compared to the female, and there was no statistical significance of contamination between the two groups. But a study by Muhadi et al. showed that males had more contaminated white coats compared to females which were not the case in our current study where more contamination was from females. We could not explain the exact cause of this difference. Most of the contaminated white coats were from students; these students account for 66.67% of the study participants. Most of them were from off-campus; this trend was similar to the study by Robati et al., where more contamination was seen off-campus compared to in-campus [14]. Furthermore the isolated* E. coli* were obtained from students' white coats; all these students were staying off-campus and were rotating in nonsurgical departments. Furthermore most of the contaminated white coats were from nonsurgical departments which contributed about 55.56% of study participants (Figure 2). Most of the study participants were stationed at inpatients department (83.33%). There was statistical significant difference in bacterial contamination of white coats of those who work in nonsurgical department compared to surgical department (*P* value < 0.001). This might be explained by the fact that most doctors and medical students in this specialty do scrubbing and use mostly theatre gown and then leave their whites coat less contaminated. They wear them less often compared with nonsurgical department where most of the time they put on white coat, hence more time to facilitate contamination (Table 2). We included 20 white coats which were not worn from laundry unit as a control and none of them were found to harbour any bacterial contaminants. This means our results are not from contamination but the real scenario of contamination which takes place in our health care settings.

Due to significant number of contaminants of clinical white coats for both students and medical doctors, there is need for revisiting the regulation of infection prevention control (IPC) in our set-up. IPC measures include practising appropriate hand hygiene and glove usage which must be a major contributor toward patients safety and reduction in cross contamination between health care provider and patients [13]. The significant number of pathogenic bacteria such as* E. coli* calls for urgent response and strictness of IPC measures to keep our patients free from nosocomial infections. Our current study showed significant percentage of bacterial contamination of white coats which supported the USA study by Collins and our results were against UK study by Burden et al. which concluded that white coats may not be major culprit in the spread of nosocomial infection [15, 16]. The existing difference may be explained by the different environment settings, where in the UK the hygienic hospital environment, isolation of infectious diseases from noninfectious ones, less populated hospitals, and organized hospital laundering services may not contribute much to the bacterial contamination of white coats.

In Tanzania, most of our wards are mixed, with both infectious and noninfectious patients, insufficient hand washing points, inadequate reinforcement of IPC regulations, congestion, and no organized laundering services for staff white coats. In developing countries like Tanzania, paucity of data exists on the incidence nosocomial infection as well as clinical hygiene related to medical doctors white coats [17]. World Health Organization (WHO) insisted on hand washing as a cornerstone to control nosocomial infection globally, but its practices vary across regions. Wearing of white coats by medical professional is accepted practice but when, where, and how we wear and wash them vary among individuals and even between different institutions [10, 17]. The existing difference may bring huge difference on its role as a vector for diseases spreading. In Tanzanian wards, we mix up both infectious and noninfectious cases, insufficient hand washing points, running out of soaps and other washing solvents, and congestion with no organized laundering services for staff white coats; therefore more chances for white coats contamination will be high. Therefore we encourage combined effort between WHO, Ministry, and IPCs committees to join their effort towards practices of hygienic procedures [17, 18]. The fatal outbreak of Ebola virus disease in West Africa could be contained if infection prevention control of hemorrhagic fever was strictly followed. We cannot afford the costs for neglecting proven prevention measures for control of hospital infections [19]. The scenario of contamination in this study might be almost similar to other countries in Africa with the same settings of facilities as ours; effort should be taken to review and reinforce our infection prevention controls to reduce the burden of infectious diseases in Africa which is already a huge problem.

Clinical white coats of medical doctors and students may be contaminated with both pathogenic and nonpathogenic bacteria. Most of the study participants in the current study were storing their coat in the living room and were staying off-campus, hence more risks to the innocent community. Effort should be made to discourage usage of white coats outside clinical areas such as canteen, supermarkets, conference halls, and chapels. Lastly, medical care providers should change their white coats as frequently as they can to reduce the chance of contamination.

## Figures and Tables

**Table 1:** Social demographic characteristics of participants (*N* = 180).

Variable	*N* (%)
Sex	
Male	118 (65.6)
Female	62 (34.4)
Staff position	
Medical doctors	60 (33.33)
Medical students	120 (66.67)
Department	
Surgical	80 (44.44)
Nonsurgical	100 (55.56)
Duty station	
Inpatients	150 (83.33)
Outpatients	30 (16.67)
White coat storage after working hours	
Hospital	28 (15.56)
Home/hostel	152 (84.44)
Wearing clinical coats outside clinical area	
Yes	8 (4.44)
No	172 (95.56)

**Table 2:** Risk factors associated with the detection of pathogens in clinical coats among study participants at KCMC, Moshi, Tanzania, in 2014.

Variables	Pathogens detected		Crude OR (95% CI)	*P*	Adjusted OR^*α*^ (95% CI)	*P* ^*μ*^
	Yes	No				
	*n* (%)	*n* (%)				
Gender						
Female	44 (31.4)	18 (45.0)				
Male	96 (68.6)	22 (55.0)	1.8 (0.9–3.7)	0.114	1.4 (0.1–3.1)	0.354
Position (level)						
Medical student	94 (67.1)	26 (65.0)				
Medical doctor	46 (32.9)	14 (35.0)	0.9 (0.4–1.9)	0.800	1.3 (0.6–2.8)	0.576
Area of residence						
Off-campus	114 (81.4)	30 (75.0)				
In-campus	26 (18.6)	10 (25.0)	0.7 (0.3–1.6)	0.372	1.1 (0.4–2.7)	0.903
Working specialty						
Surgical	74 (52.9)	6 (15.0)				
Nonsurgical	66 (47.1)	34 (85.0)	0.2 (0.1–0.4)	<0.001	0.2 (0.1–0.5)	<0.001
Duty station						
Inpatients	112 (80.0)	38 (95.0)				
Outpatient	28 (20.0)	2 (5.0)	4.8 (1.1–20.9)	0.039	3.2 (0.7–14.9)	0.132
Days of worn coat since last washing						
<3 days	94 (67.2)	26 (65.0)				
3–7 days	36 (25.7)	14 (35.0)	0.7 (0.3–1.5)	0.376	0.6 (0.2–1.3)	0.205
>7 days	10 (7.1)	0 (0.0)	—	—	—	—
Wearing a white coat outside clinical areas						
Yes	6 (4.3)	2 (5.0)				
No	134 (95.7)	38 (95.0)	1.2 (0.2–6.1)	0.847	1.3 (0.2–7.3)	0.802
Location for coat storage						
Hospital area	22 (15.7)	6 (15.0)				
Home/hostel	118 (84.3)	34 (85.0)	0.9 (0.4–2.5)	0.912	0.6 (0.2–1.8)	0.412

RR = relative risk; CI = confidence interval.

^*μ*^Estimated from the logistic regression model with Wald type *P* value.

^*α*^Adjusted for gender, working specialty, and duty station.

**Table 3:** Organism isolated from white coats (*N* = 132).

S. number	Organism(s)	Numbers of isolates	Percentage of isolates
1	*Staphylococcus aureus*	120	90.91%
2	*Pseudomonas aeruginosa*	9	6.82%
3	*Escherichia coli*	3	2.27%
	Total	132	100%
